# COMPARATIVE ANALYSIS OF LATE RESULTS OF CERVICAL ESOPHAGOGASTRIC ANASTOMOSIS BY MANUAL AND MECHANICAL SUTURE IN PATIENTS SUBMITTED TO ESOPHAGEAL MUCOSECTOMY THROUGH ADVANCED MEGAESOPHAGUS

**DOI:** 10.1590/0102-672020190001e1462

**Published:** 2019-12-20

**Authors:** José Luis Braga de AQUINO, Vania Aparecida LEANDRO-MERHI, José Alexandre MENDONÇA, Elisa Donalisio Teixeira MENDES, Conceição de Maria Aquino Vieira CLAIRET, Leonardo Oliveira REIS

**Affiliations:** 1 Graduate Program in Health Sciences, Pontifical Catholic University of Campinas, Campinas, SP, Brazil.

**Keywords:** Esophageal achalasia, Anastomosis, surgical, Suture techniques, Acalásia esofágica, Anastomose cirúrgica, Técnicas de sutura

## Abstract

**Background:**

Among the anastomoses of the gastrointestinal tract, those of the esophagus are of special interest due to several anatomical or even general peculiarities.

**Aim:**

Evaluate retrospectively the results comparing mechanical vs. manual suture at cervical esophagogastric anastomosis in megaesophagus treatment.

**Methods:**

Were included 92 patients diagnosed with advanced megaesophagus with clinical conditions to undergo the surgery. All underwent esophageal mucosectomy, performing anastomosis of the esophagus stump with the gastric tube at the cervical level. In order to make this anastomosis, the patients were divided into two groups: group A (n=53) with circular mechanical suture, lateral end; group B (n=39) with manual suture in two sides, lateral end. In the postoperative period, an early evaluation was performed, analyzing local and systemic complications and late (average 5.6 y) analyzing deglutition.

**Results:**

Early evaluation: a) dehiscence of esophagogastric anastomosis n=5 (9.4%) in group A vs. n=9 (23.0%) in group B (p=0.0418); b) stenosis of esophagogastric anastomosis n=8 (15.1%) in group A vs. n=15 (38.4%) in group B (p=0.0105.); c) pulmonary infection n=5 (9.4%) in group A vs. n=3 (7.6%) in group B (p=1.0000.); d) pleural effusion n=5 (9.4%) in group A vs. n=6 (15.4%) in group B (p<0.518). Late evaluation showed that 86.4-96% of the patients presented the criteria 4 and 5 from SAEED, expressing effective swallowing mechanisms without showing significant differences among the groups.

**Conclusion:**

Cervical esophagogastric anastomosis by means of mechanical suture is more proper than the manual with lower incidence of local complications and, in the long-term evaluation, regular deglutition was acquired in both suture techniques in equal quality.

## RESUMO

**Racional:**

Das anastomoses do trato gastrointestinal, as do esôfago têm especial interesse devido às varias peculiaridades anatômicas e mesmo sistêmicas.

**Objetivo:**

Avaliar retrospectivamente os resultados comparando a sutura mecânica e manual na anastomose esofagogástrica cervical no tratamento do megaesôfago.

**Métodos:**

Foram estudados 92 pacientes com diagnóstico de megaesôfago avançado com condições clínicas de serem submetidos à operação. Todos foram submetidos à mucosectomia esofágica, sendo realizada anastomose do coto esofágico com o tubo gástrico no nível cervical. Para a realização desta anastomose, foram divididos em dois grupos: grupo A (n=53) com sutura mecânica circular terminolateral; grupo B (n=39) com sutura manual em dois planos terminolateral. No período pós-operatório foi realizada avaliação precoce, com análise das complicações locais e sistêmicas, e tardia (média 5,6 anos) com análise da deglutição.

**Resultados:**

Avaliação precoce: a) deiscência da anastomose esofagogástrica, n=5 (9,4%) no grupo A vs. n=9 (23,0%) no grupo B (p=0.0418); b) estenose da anastomose esofagogástrica n=8 (15,1%) no grupo A vs. n=15 (38,4%) no grupo B (p=0.0105); c) infecção pulmonar n=5 (9,4%) no grupo A vs. n=3 (7,6%) no grupo B (p=1.000); d) derrame pleural n=5 (9,4%) no grupo A vs. n=6 (15,4%) no grupo B (p<0.518). A avaliação tardia demonstrou que 86,4 a 96,0% dos pacientes apresentaram critérios 4 e 5 de SAEED, demonstrando deglutição efetiva e sem diferença significante entre os grupos.

**Conclusão:**

A anastomose esofagogástrica pela sutura mecânica é mais adequada que a manual com pequena incidência de complicações locais e, na avaliação em longo prazo, a deglutição demonstrou ser adequada em ambos os grupos e com qualidade semelhante.

## INTRODUCTION

Among the anastomoses of the gastrointestinal tract, those of the esophagus are of special interest due to several anatomical or even general peculiarities, which distinguish them from other segments of the digestive tract ^5^ ^,^ ^14^ ^,^ ^15^ ^,^ ^18^. Hence, the anastomotic dehiscences in this organ appear with a higher incidence, prolonging the permanence of patients in hospitals as well as increasing hospital costs, causing greater suffering for the patients and showing a relationship with stenosis, which is another obstacle that follows the esophageal surgery^5^ ^,^ ^18^. On the other hand, the mechanical suture demonstrating more safety, precision and fastness, predisposes to a lower incidence of anastomotic fistula, as it has been demonstrated in the literature, both in benign and malignant diseases, being able to improve the quality of life ^2^ ^,^ ^4^ ^,^ ^5^ ^,^ ^9^ ^,^ ^20^ ^,^ ^21^ ^,^ ^30^. This preference can be justified by less ischemia, less extensive tissue necrosis and a more pronounced neoangiogenesis, as has been demonstrated in experimental studies^4^ ^,^ ^15^ ^,^ ^18^ ^,^ ^32^. 

In advanced megaesophagus of chagasic origin the disease damages the contractility of the organ due to plexular denervation impairing deglutition, with the consequent malnutrition. Besides, due to the stasis that occurs over the years, it may also induce the development of cancer^5^ ^,^ ^17^.

As it has been demonstrated by several authors for many years, the best therapy is esophagectomy without thoracotomy^13^, as it acts directly in the physiopathology of this disease, being the transmediastinal technique proposed by Pinotti the most used^5^ ^,^ ^8^ ^,^ ^22^ ^,^ ^23^. More recently with the advent of minimally invasive surgery, this resection can be performed by videolaparoscopy^12^ ^,^ ^29^. In a more critical analysis; however, it has been demonstrated that transmediastinal esophageal resection is not free from complications which may contribute to greater morbidity in the postoperative^8^ ^,^ ^10^ ^,^ ^19^. This may occur, as the advanced megaesophagus presents periesophagitis leading to the adherence to mediastinal structures predisposing and complications.

This fact stimulated Aquino et al.^3,6^ to propose the technique of esophageal mucosectomy with preservation of the muscular layer and transposition of the stomach into the muscular layer of the esophagus for the reconstruction of the digestive transit and anastomosis of the stomach with the stump of the cervical esophagus by the technique of manual suture. Thus, a lower rate of complications was observed in relation to esophagectomy without thoracotomy, because it did not transgress the mediastinum during dissection of the esophagus.

In another paper, Aquino et al.^1^ demonstrated the advantages of mechanical suture in relation to manual cervical esophagogastric anastomosis due to the small frequency of anastomotic fistula in patients with advanced megaesophagus but submitted only to transmediastinal esophagectomy.

Consequently, the idea to perform this study was emerged, comparing the manual suture and its mechanics to the level of cervical esophagogastric anastomosis in patients submitted to esophageal mucosectomy through advanced megaesophagus.

Therefore, this study aims to demonstrate if the mechanical suture presents advantages over the manual one for attempting to minimize the anastomotic dehiscence and thus provide an earlier and more proper deglutition with potential improvement of the nutritional status.

## METHOD

This study was approved by institutional ethics committee under number 1.277.805

### Casuistic

Between January of 1996 to December 2017, 92 patients with advanced megaesophagus were retrospectively evaluated in the Department of Thoracic Surgery, Celso Pierro Hospital, Faculty of Medicine of the Pontifical Catholic University of Campinas, Campinas, SP, Brazil. Among the studied patients, there was a predominance of male patients in 75% (n=69) with the age ranging from 23 to 63 years old (mean- 48.5 y).

### Clinical data

All patients reported progressive dysphagia from solids to liquids and weight loss with variable time from 5-15 years, with 85.9% (n=79) reporting more frequent intermittent regurgitation in the last six to 24 months. From the total 68.4% (n=63) were smokers of 20 cigarettes/day for a variable time of 12- 26 years, and 55.4% (n=51) reported be distillate drinkers of 2-5 dose/day for 11-15 years.

Immunofluorescence for Chagas’ disease was positive in 90.2% (n=83). The clinical and nutritional assessment demonstrated weight loss in 20.6% (n=19), losing more than 10% of their ideal weight and submitted to nasoenteral probe for a variable time of 18-33 days prior to the surgical procedure.

### Diagnostic assessment

#### Radiology

In all patients the contrasted esophageal radiological study showed megaesophagus grade III in 41.3% (n=38) and grade IV in 58.7% (n=54), according to the classification of Rezende et al.^25^.

#### Endoscopy

In all patients the exam showed an increase in the diameter of the organ with distal esophagus mucosa presenting grade A to C esophagitis in Los Angeles Classification and without evidence of neoplasia in any one. 

#### Manometry

Was performed in 30.4% (n=28) demonstrated in all aperistalsis of the esophagus body and decreased relaxation of the esophageal lower sphincter.

#### Surgical technique

All patients underwent esophageal mucosectomy with preservation of the muscular layer, according to the technique standardized by Aquino et al.^3^ ^,^ ^6^. In doing the cervical anastomosis the patients were divided into two groups, A and B. 

In group A (n=53) mechanical suture used circular DHC 29 mm device; for this anastomosis the ogive was fixed in the stump of the cervical esophagus and it was introduced through the anterior side of the stomach and attached to the ogive; the anterior side of the stomach, through which the apparatus was introduced, was sutured with the mechanical technique with a 75 mm linear suture. 

In group B (n=39) manual two planes with 3-0 Vicryl^®^ suture was, being the first one continuous and complete in the stomach and esophagus, and the second in separate stitches, seromuscular in the stomach and muscular in the esophagus

### Postoperative assessment 

#### Clinical complications

Especially focused in cardiovascular, respiratory and infectious complications the diagnosis was based on the daily clinical progress with laboratory and imaging exams, when necessary.

#### Local complications

They were mainly related to dehiscence and stenosis of the anastomosis of the cervical esophagus with the gastric tube with consequent fistula, the diagnosis was clinical due to the exit of gastric and/or salivary secretion by the cervical region until generally the 7^th^ postoperative day. From that day on, without any evidence of fistula, a contrast X-ray was performed to evaluate if there was output of contrast by the anastomosis. When this did not occur, the oral diet was initiated. In relation to anastomosis stenosis, the diagnosis was clinical due to the symptom of dysphagia, especially from the 30^th^ postoperative day and verified by the X-ray contrast at the level of the anastomosis and upper digestive endoscopy, to indicate in both examinations whether the anastomosis diameter decreased.

#### Deglutition

In the long-term follow-up deglutition was evaluated at 1, 3, 5 and 10 years postoperatively, based on Saeed et al.^28^ criteria: 0=no swallowing; 1= swallows liquid with difficulty, but does not swallow neither pasty nor solids; 2= swallows normal liquid, pasty with difficulty, and does not swallow solids; 3= swallows liquid and pasty normally, but swallows solids with difficulty; 4= swallows liquid and pasty normally, eventual difficulty to swallow solids; 5= normal swallowing. 

Other symptoms that could be related to the surgical procedure performed, mainly regurgitation, were also assessed. 

### Statistical analysis

For comparison, the chi-square test or the exact fisher test, were used when necessary, with a significance level of 5%.

## RESULTS

In the early evaluation up to 30 postoperative days, 14 patients (15.2%) presented fistula of the esophagogastric anastomosis at the level of the cervical region, being significantly smaller in group A (Table 1). To one patient from this group was indicated early reoperation because presented the leak on the 2^nd^ postoperative day, having extensive drainage of the cervical and mediastinal region; this patient had a good evolution. In the other 13 patients in which anastomosis fistula occurred between the 4^th^ and 7^th^ postoperative days, the treatment was conservative with local drainage of the cervical region and nutritional support with enteral diet by jejunostomy, with the fistula closing between the 14^th^ and 23^rd^ day of postoperative period. Thus, since there was no further digestive secretion through the cervical region, a contrasted X-ray was required. Showing no signs of contrast extravasation to the anastomosis level, oral diet initially liquid with a progressive replacement for pasty and solid, according to the patient’s acceptance, was introduced^16^. This orientation was also performed in the other 78 patients who did not present anastomotic fistula, having the oral diet from the 7^th^ postoperative day after confirming that the contrasted X-ray did not indicate extravasation. All accepted the diet well, in a progressive way. 

**TABLE 1:** Comparative analysis of early postoperative complications between suture groups

	Group A (n=53)	Gruop B (n=39)	p
	n (%)	n (%)	
Anastomotic fistula	5 (9.4)	9 (23.1)	0.0418 Chi2
Anastomotic stenosis	8 (15.1)	15 (38.5)	0.0105 Chi2
Pulmonary infection	5 (9.4)	3 (7.7)	1.0000 F
Pleural effusion	5 (9.4)	6 (15.4)	0.5182 F
Death	0 (0.0)	1 (2.6)	NC

A=mechanical suture; B=manual suture; *p*=Fisher’s exact test; Chi2=Chi-square test; NC-not calculated

Eight patients (8.6%) between the 5^th^ and 9^th^ postoperative day presented pulmonary infection, without significant differences between the groups (Table 1). With the exception of one patient from group B who evolved to death caused by septic shock, all the remaining had positive progress. 

Small to medium volume pleural effusion was present in 11 (11.9%) patients, without significant differences between groups (Table 1). In five (n=3 from group A and n=2 from group B) it was necessary to drain the thorax leading to a positive outcome; in the remaining patients who presented this complication, the progress was also positive with the proper conservative treatment. 

Between the 30^th^ and 48^th^ postoperative day, 23 patients (25%) began to present the symptom of advanced progressive dysphagia, confirming stenosis of the esophageal anastomosis at the cervical level by contrasted X-ray and upper digestive endoscopy, being significantly greater in group B. All patients underwent endoscopic dilation ranging from 4-15 sessions with a positive progress.

The mid and long-term assessment were performed in 71 patients, 69.8% (n=37) from group A and 87.1% (n=34) from group B, with time ranging from 1 to 10 years (mean 5.6 y).

In relationship to deglutition, 86.4% to 96.0% of the patients presented the criteria 4 to 5 from Saeed et al.^28^ with assessment time ranging from 1 to 10 years without presenting significant differences between the two groups (Table 2).

**TABLE 2:** Descriptive analysis and comparison of late assessment between suture groups

Late assessment	A		B		p	
	n %		n %			
1 YEAR -criteria 0 1 2 3 4 5 3 YEAR-criteria 0 1 2 3 4 5	n=37 0 0 0 3 7 27 n=37 0 0 0 3 6 28	0.0 0.0 0.0 8.1 18.9 73.0 0.0 0.0 0.0 8.1 16.2 75.7	n=34 0 0 0 3 7 24 n=34 0 0 0 1 11 22	0.0 0.0 0.0 8.8 20.6 70.6 0.0 0.0 0.0 2.9 32.4 64.7	1.0000 0.2354	F F
5 YEAR-criteria 0 1 2 3 4 5	n=37 0 0 0 5 10 22	0.0 0.0 0.0 13.5 27.0 59.5	n=34 0 0 0 4 5 25	0.0 0.0 0.0 11.8 14.7 73.5	0.3753	F
10 YEAR-criteria 0 1 2 3 4 5	n=37 0 0 0 3 11 23	0.0 0.0 0.0 8.1 29.7 62.2	n=34 0 0 0 4 9 21	0.0 0.0 0.0 11.8 26.5 61.8	0.8787	F

A=mechanical suture and B=manual suture; p=Fisher’s exact test

The intermittent and sporadic regurgitation that was present with not negligible incidence, did not present significant differences between the groups and was controlled with proper food orientation (Table 3). Is evident in this table that two patients, one from each group, presented Barrett’s esophagus in the esophageal stump without presenting statistical significance, having the diagnosis been done in the 3^rd^ and 5^th^ postoperative year with these patients being examined periodically with endoscopy. Most patients from both groups reported weight gain.

**TABLE 3:** Late comparative assessment of postoperative between suture groups

	Group A (n=37)		Group B (n=34)		p
	n	%	n	%	
Regurgitation	9	24.3	9	26.5	0.8355 Chi2
Barrett’s esophageal stump	1	2.7	1	2.9	1.0000 F

A=mechanical suture; B=manual suture; p=Fisher exact test; Chi2=Chi-square test

## DISCUSSION

Throughout the world surgical history, the stenoses, fistulas, and dehiscences resulting from anastomoses between viscera of the digestive system are justified by the fear of their presence, since they often evolve to excessive morbidity and not infrequently to death. Thus controversies over the best type of suture remains to the current days^1^ ^,^ ^5^ ^,^ ^20^ ^,^ ^21^.

Because the esophagus is the segment of the digestive tract that presents a higher incidence of dehiscences of anastomoses by the peculiarities already mentioned, it was necessary to use mechanical suture for the advantages it offers and reducing the frequency of this complication, especially in the cervical esophagus, as demonstrated in several series^1,5, 8,14,18,19,30^. This fact became very evident in our study, since the patients from the mechanical suture group presented a significantly smaller incidence of dehiscence of the esophagogastric anastomosis in relation to the manual suture group, after esophageal mucosectomy.

Another fact to be considered is that the mechanical suture, for being double and inverted, could predispose stenosis of the esophagogastric anastomosis by up to three to five times in relation to manual, as has been demonstrated by several authors^1^ ^,^ ^4^ ^,^ ^15^ ^,^ ^19^ ^,^ ^20^ ^,^ ^31^. This is justified by the fact that most of the patients in related studies underwent esophagectomy due to cancer and therefore, presented a smaller diameter of the cervical esophageal stump to be anastomosed, so it became imperative to use staplers of smaller sizes. This fact was well evidenced a few years ago by Wong et al.^33^, when they correlated the staplers’ diameter with the incidence of stenosis. These authors demonstrated that when they used devices with a diameter of 25 mm, the incidence of stenosis was 25% and decreased to 12% with the use of staplers from 29-33 mm. Recently, Honda et al.^14^ have also demonstrated in a literature review comparing manual with mechanical suture in 1,407 patients undergoing esophageal anastomosis, a very adequate correlation between the stapler diameter and the degree of stenosis of the esophagogastric anastomosis.

Because the lumen of the esophageal stump to be anastomosed is greater in the advanced megaesophagus, it is possible to use devices with greater diameters, hence, reducing the incidence of stenosis. This was also evident in our study, since although this complication was present in patients from both groups, it was significantly smaller in patients who underwent mechanical suture, and this can be justified for having been used in all the circular device of number 29 mm.

Another fact to remember is that of the 23 patients who presented stenosis of esophageal anastomosis, 14 (60.8%) had previously fistula, and the relationship between anastomotic dehiscence and stenosis is frequent due to the fibrosis that occurs at the suture line after the anastomotic fistula closure, as has been demonstrated in several series^1^ ^,^ ^5^ ^,^ ^11^ ^,^ ^14^ ^,^ ^18^ ^,^ ^19^. Although there was a delay in the normal swallowing of these patients, they presented a positive progress after the endoscopic dilations and they reported being satisfied with the surgical procedure.

The performed technique of the esophageal mucosectomy with conservation of the muscular layer with the transposition of the stomach inside this layer was of great validity, as it avoided to transgress the mediastinum and thus prevented the potential lesions of the noble structures present there. Nevertheless, these complications are mentioned when the transmediastinal esophagectomy is used, and although not common, when they are actually present, there is a great potential for morbidity^8^ ^,^ ^19^ ^,^ ^22^ ^,^ ^23^. This situation has been proven in the patients of our series because none of them, from both groups, presented such complications at the mediastinal level, as the early assessment demonstrated. Nevertheless, some evolved to pleural effusion, but of low incidence in both groups and easily exited by conservative treatment and/or thoracic drainage.

Because esophageal mucosectomy is a major surgery and most patients from both groups are long-time smokers, it can be justified that eight patients presented pulmonary infection, and although there was specific clinical treatment, one in group B evolved to death from septic shock. This has also been demonstrated in various series, when esophagectomy is performed either by benign or malignant diseases^5^ ^,^ ^8^ ^,^ ^18^ ^,^ ^19^ ^,^ ^23^ ^,^ ^30^.

In the long-term evaluation with the average follow-up of 5.6 years in 71 patients, all of them reported being satisfied with the surgery, as 86% to 96% of them from both groups presented total recovery of swallowing with 4 and 5 Saeed et al. criteria^28^, which caused most of the patients to report weight gain and encourage them to return to their routine work activities. Although dysphagia for solid foods was present in some patients of both groups, this was not only intermittent, but also of low frequency and without significant differences between the groups.

The regurgitation was around 25% in both groups and was resolved after proper diet. This change is described in up to 50% in patients who undergo gastric transposition to replace the esophagus either by benign or malignant conditions^3^ ^,^ ^4^ ^,^ ^5^ ^,^ ^6^ ^,^ ^8^ ^,^ ^18^ ^,^ ^19^ ^,^ ^20^ ^,^ ^23^ ^,^ ^30^. This is usually a consequence of gastric stasis and if a proper diet is followed, the patients usually adapt well without compromising the usual activities.

Another fact to be remembered is that one patient from each group evolved in the late postoperative period with Barrett’s esophagus in the esophageal stump. This change is usually a consequence of acid reflux and bile of the stomach transposed due to stasis, as has been demonstrated by some authors^24^ ^,^ ^26^ ^,^ ^27^. Although this complication was of low incidence, it needs to be considered due to the possibility of this compromised epithelium evolve to adenocarcinoma, as has already been demonstrated^17^ ^,^ ^27^. Hence, it is important to carry out a long-term follow-up with periodic digestive endoscopies, as recommended in these two patients in our study, or even the prolonged use of proton pump inhibitors to minimize reflux and its complications^24^ ^,^ ^27^.

A doubt that has always been present is that what could occur in the long-term assessment with these patients undergoing esophageal mucosectomy, whether the muscular layer of the esophagus at the level of the mediastinum could evolve to fibrosis with the consequent compression of the stomach and thus compromise the progress. The postoperative analysis, with an average follow-up time of 5.6 years in 71 patients showed that all of them progressed well, without presenting any symptoms that would suggest gastric compression.

And this fact was already evident in other studies performed by us, but with a smaller number of patients and with the cervical esophagogastric anastomosis performed only with manual suture and with imaging tests by thorax tomography, evidencing that the permanence of the muscular layer in the posterior mediastinum does not seem to have compromised the gastric transposition for reconstruction of the digestive tract after esophageal mucosectomy^3^ ^,^ ^7^.

## CONCLUSION

Esophageal mucosectomy with preservation of the muscular layer for the treatment of advanced megaesophagus is adequate procedure, due to the low rate of pleuropulmonary complications, absence of mediastinal complications and adequate recovery of swallowing in the majority of long-term follow-up. The mechanical suture of the cervical esophagogastric anastomosis is more adequate because it presents significant lower incidence of anastomotic complications than the manual technique.
